# Multioxide Nanomaterials Zn, Cu, and Mn: Combining Nanozyme and Photocatalyst Functions for Environmental Applications

**DOI:** 10.1021/acsomega.4c08949

**Published:** 2025-05-14

**Authors:** Julia Matysik, Olga Długosz, Marcin Banach

**Affiliations:** † CUT Doctoral School, Faculty of Chemical Engineering and Technology, 49571 Cracow University of Technology, Warszawska St. 24, 31-155 Cracow, Poland; ‡ Faculty of Chemical Engineering and Technology, 49571 Cracow University of Technology, Warszawska St. 24, 31-155 Cracow, Poland

## Abstract

The study analyses nanotechnology advances in the synthesis of catalytic nanozymes that mimic enzymatic properties but are also inorganic nanomaterials. It compares Zn–Mn–Cu multioxide and Mn–Cu multioxide nanomaterials in terms of the role of nanozymes and photocatalysts in the decomposition of trypan blue dye. The study showed that Zn–Mn–Cu multioxide nanoxide has activity as a peroxidase-like nanozyme (100 mUnit/mL) and achieves 75% dye degradation as a photocatalyst under UV light. At the same time, Mn–Cu multioxide shows enhanced photocatalysis under visible light (45% degradation), consistent with its biocatalytic-like hydrolysis mechanism. In addition, both materials showed biocatalytic-like activity in the degradation of trypan blue dye (25%), without an additional light source. Further investigation of these dual nanozyme activities may reveal novel solutions for environmental photocatalysis.

## Graphical abstract

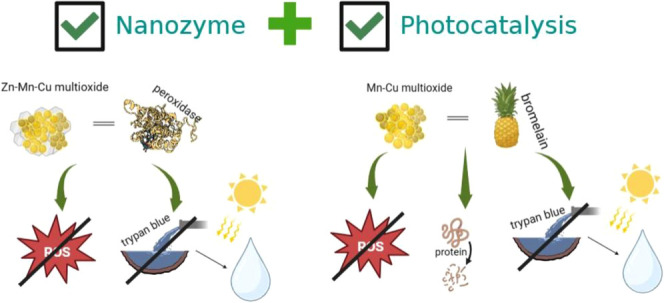

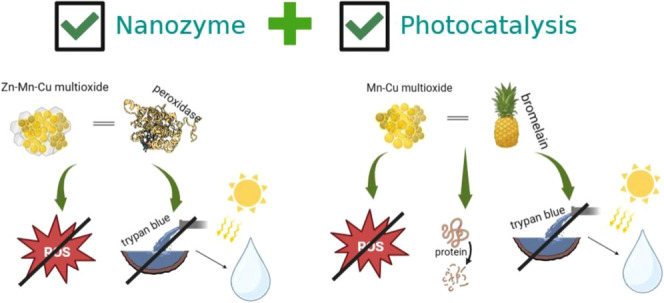

## 1 Introduction

Advances in nanotechnology have paved the way for the synthesis of nanoparticles with adapted properties, including shape, size, and surface modifications, thereby affecting their chemical and physical properties.^1^ Nanozymes are one type of material with catalytic properties, gaining notable attention from researchers.^2–4^

Nanozymes are nanomaterials that exhibit properties that mimic enzyme-mediated biocatalysis. Thanks to their physicochemical properties, combining the characteristics of inorganic materials and enzyme-mimicking activity, while preserving the nature of the nanomaterial formula.^5^ Natural enzymes are often limited by their thermostability and activity in a specific pH range, making them vulnerable to environmental changes.^6,7^ On the contrary, nanozymes can operate effectively outside the typical physiological parameters of temperature and environmental pH, without denaturing or significantly impairing their catalytic activity. This wider range of stability and enzyme-mimicking functionality makes nanozymes an alternative between photocatalytic nanomaterials and systems with immobilized proteins.^8,9^

Nanozymes have the ability to mimic natural enzymes, allowing them to reduce reactive oxygen species,^10,11^ whether to reduce the effects of oxidative stress on cells.^12^ Currently known nanozymes mimic the activities of enzymes from the oxidase or hydrolase groups.^5^ In particular, nanozymes with the character of enzymes from oxidoreductase groups such as superoxide dismutase (SOD), catalase (CAT), peroxidase (POD), or glutathione peroxidase (GPX) are distinguished.^4,13^ An essential element in defining a material as a nanozyme is the determination of the catalytic activity, which should result from mechanisms similar to those of enzymes.^14^

Nanozymes can be tailored for specific reactions, enabling their use as biosensors^15,16^ and in other biomedical applications such as bioimaging.^17,18^ Currently, nanozymes are being investigated as reactants for the detection of heavy metals^5^ or catalysts,^19^ and their use for environmental applications is only being developed.^6,20^ One environmental application is the use of nanomaterials as photocatalysts in decomposition reactions of dyes and organic substances.^21,22^ Photocatalyst materials based on copper oxides,^23^ zinc oxides,^22^ or manganese oxides^24^ can be found in the literature, but trioxide systems of such nanomaterials with enzymatic activity have not been studied for use in photocatalysis. Multioxide materials, in studies of nanozymes, are most often used as glucose sensors, and studies of their activity are not aimed at using biocatalysis.^25^ Our previous work explored the effect of metallic additives on the formation of nanozymatic materials, offering mechanistic insights into the emergence of nanozyme-like activity.^26^

Therefore, comprehensive studies on the enzymatic mimicry reactions of nanozymes (not only related to peroxidase activity) and studies about the use of nanozymes in the dual role of photo- and biocatalysts are lacking in the literature. This research presents a significant advancement in the field of nanozymes by exploring the dual functionality of Zn–Mn–Cu and Mn–Cu multioxide nanomaterials as both enzyme mimics and photocatalysts. The novelty lies in the integration of biocatalytic and photocatalytic properties within a single material system, which has not been comprehensively studied before, particularly for the degradation of environmental pollutants, such as dyes. By leveraging the synergistic effects of biologically active metal oxides, such as manganese, copper, and zinc, the study addresses a critical gap in the literature, bridging the divide between traditional enzymatic systems and photocatalytic nanomaterials. This innovative approach allows the materials to operate effectively, mimicking the activity of specific enzyme groups such as oxidoreductases and hydrolases while also exhibiting high photocatalytic efficiency under ultraviolet and visible light. The findings not only advance the fundamental understanding of the reaction mechanisms of nanozymes but also highlight their potential for practical applications in environmental remediation, emphasizing the importance of developing sustainable and adaptable solutions for tackling complex environmental challenges.

The aim of the present study was to obtain and compare the activity of Zn–Mn–Cu and Mn–Cu multioxide nanomaterials with dual nanozyme and photocatalytic properties. The materials were characterized in terms of their enzymatic properties and their activity in the photocatalytic decomposition of the aqueous model pollutant trypan blue. In order to investigate their enzymatic activity, the activity toward the ABTS radical was determined, proteolytic assays were performed with casein degradation, and peroxidase activity in H_2_O_2_ decomposition was determined, and then a possible biocatalysis mechanism was proposed for each nanoxide, respectively. The materials contained manganese and copper oxides, due to the presence of these elements in the active centers of the enzymes. Investigations under ultraviolet light and light mimicking visible light allowed comparison of the photocatalytic activity of the nanomaterials. Biocatalysis-like studies demonstrated the usefulness of the materials in decomposing trypan blue based on the enzymatic mimicry of the nanomaterials.

## 2 Results and Discussion

### 2.1 Material Structural Analysis

The XRD analysis of the materials confirmed the presence of oxide phases suitable for each of the materials (Figure 1).

**Figure 1:**
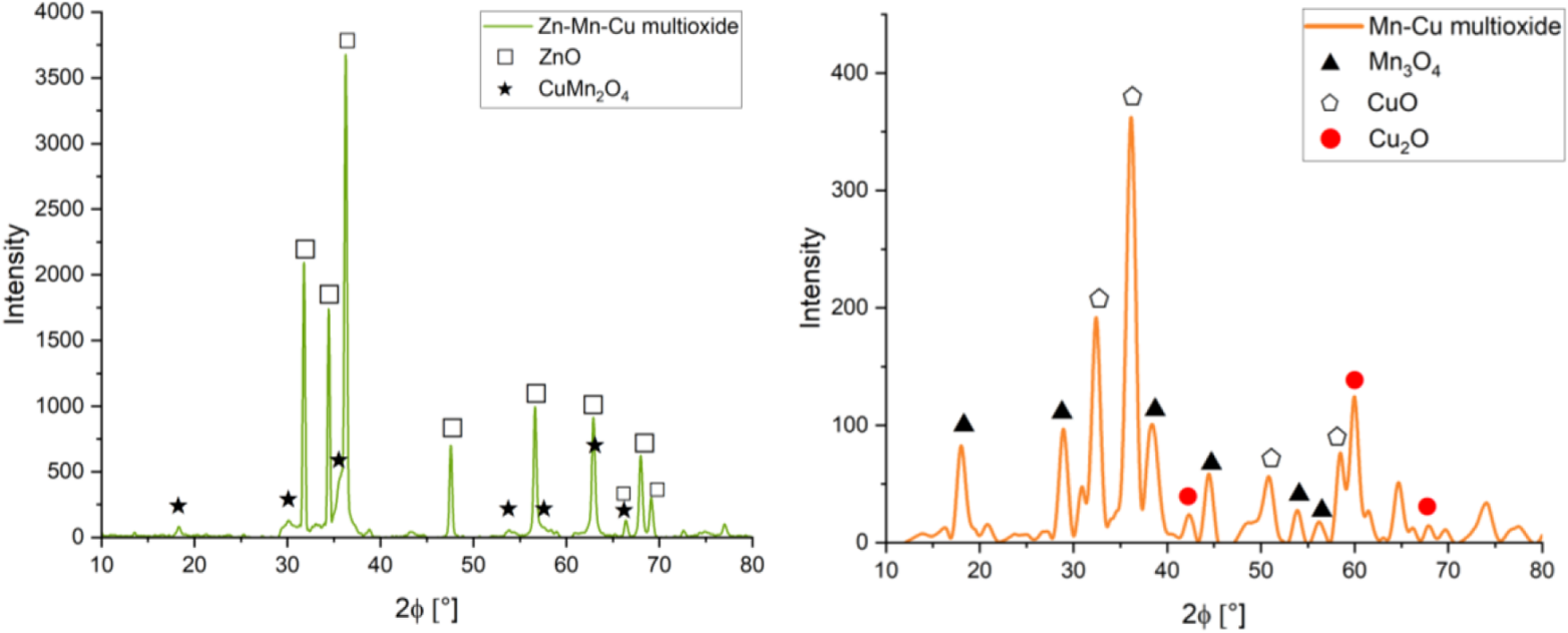
XRD patterns of (left) material Zn–Mn–Cu multioxide and (right) material Mn–Cu multioxide.

In the Zn–Mn–Cu multioxide material, ZnO (31.8, 34.46, 36.28, 47.56, 56.62, 62.88, 66.38, 67.98, and 68.1°) and Cu Mn_2_O_4_ (18.4, 30.1, 35.48, 53.84, 57.5, 62.88, and 66.38°) phases are present. These phases are reflected in the COD crystallographic data #2300116 and #1533674, respectively, for the phases. The phase ratio was 1:0.21, the elemental molar ratio was assumed as 1:0.2:0.3, and finally determined as 1:0.07:0.12 (Zn: Cu: Mn).

The Mn–Cu multioxide material contains Mn_3_O_4_ (18.01, 28.9, 38.1, 44.4, 53.9, and 56.1°), CuO (32.4, 38.6, 48.7, and 58.5), and Cu_2_O (42.32, 61.4, and 69.7°) phases, corresponding to records with the respective COD numbers #1011262, #9016105 and #1010941. The oxide phase ratio was 1:0.125:0.06. The theoretical elemental molar ratio (Mn:Cu) was expected to be 1:0.35, which was verified as 1:0.23 after XRD analysis.

Material sizes were calculated from XRD data; average crystallite phase sizes are included in the Table 1 for each phase of nanomaterials.

**Table 1:** Nanomaterials Phases Calculated Size in Nanometers Based on XRD Results

material	phase	size [nm]
Zn–Mn–Cu multioxide	ZnO	50.25
	CuMn_2_O_4_	27.70
Mn–Cu multioxide	Mn_3_O_4_	18.08
	CuO	7.85
	Cu_2_O	5.97

For the Zn–Mn–Cu multioxide material, the XRD results obtained for the ZnO phase are in agreement with those presented by Raha et al.^27^

The researchers synthesized a CuO/Mn_3_O_4_/ZnO nanomaterial system using the precipitation method to first obtain copper oxide, followed by zinc oxide and manganese oxide (II/III). The material was placed in an autoclave for the reaction, and then, the material was further calcined. In addition, the researchers characterized the separate phases of the system. The size of the ZnO phase is larger than in the study of Raha et al.,^27^ however, this may be due to the different order in which the oxides were obtained and the different methodology for synthesizing the trioxide. The spinel phase of CuMn_2_O_4_ is consistent with the XRD results presented by Sheikhzadeh and Sanjabi^28^ on copper manganese oxide films obtained by coelectrodeposition. The authors of the study particularly highlighted the presence of a peak at 18.4°, which determines the presence of the CuMn_2_O_4_ phase.

For the Mn–Cu multioxide material, the spectra for the CuO and Mn_3_O_4_ phases are also found to be similar in the study of Raha et al.,^27^ and the crystallite sizes are also similar, −10 nm for the CuO phase and 14 nm for the Mn_3_O_4_ phase. Similarly, in the study of Lee and co-workers,^29^ who obtained nanorods with CuO as the base, doped with ZnO nanoxide. The authors prepared CuO films using copper­(II) sulfate and sulfuric acid (VI), which were oxidized at 300 °C for 2 h. Then, by repeating the oxidation of zinc acetate to ZnO on the CuO surface several times, the researchers applied a layer of zinc oxide to the resulting film, and the system thus prepared was reacted in a hydrothermal process. The researchers presented XRD spectra containing distinct phases: CuO, Cu_2_O, and ZnO, which can be compared with the phases obtained for Zn–Mn–Cu multioxide and Mn–Cu multioxide materials.

In the SEM microphotographs (Figure 2), the difference in the material structures can be observed. The Zn–Mn–Cu multioxide has larger, single nanocomposite particles, on which smaller particles can be seen, forming a differentiated material structure. A difference in the shape of the nanoparticles can also be observed, with the larger elements being flat and asymmetrical and the smaller ones being more circular. The EDS analysis shows an even distribution of the zinc phase on the surface of the material, apart from the areas occupied by manganese and copper. It can be seen that Mn and Cu are visible in the same areas, confirming that the elements occur together. The Mn–Cu multioxide morphology shows more agglomerated particles of similar size with a swollen structure. The nanomaterial has rather round structures, and particle layering is visible. The microphotographs from the EDS analysis show an even distribution of both elements, manganese and copper, on the surface of the material. It is also possible to see areas occupied only by copper, which is evidence of phase separation.

**Figure 2:**
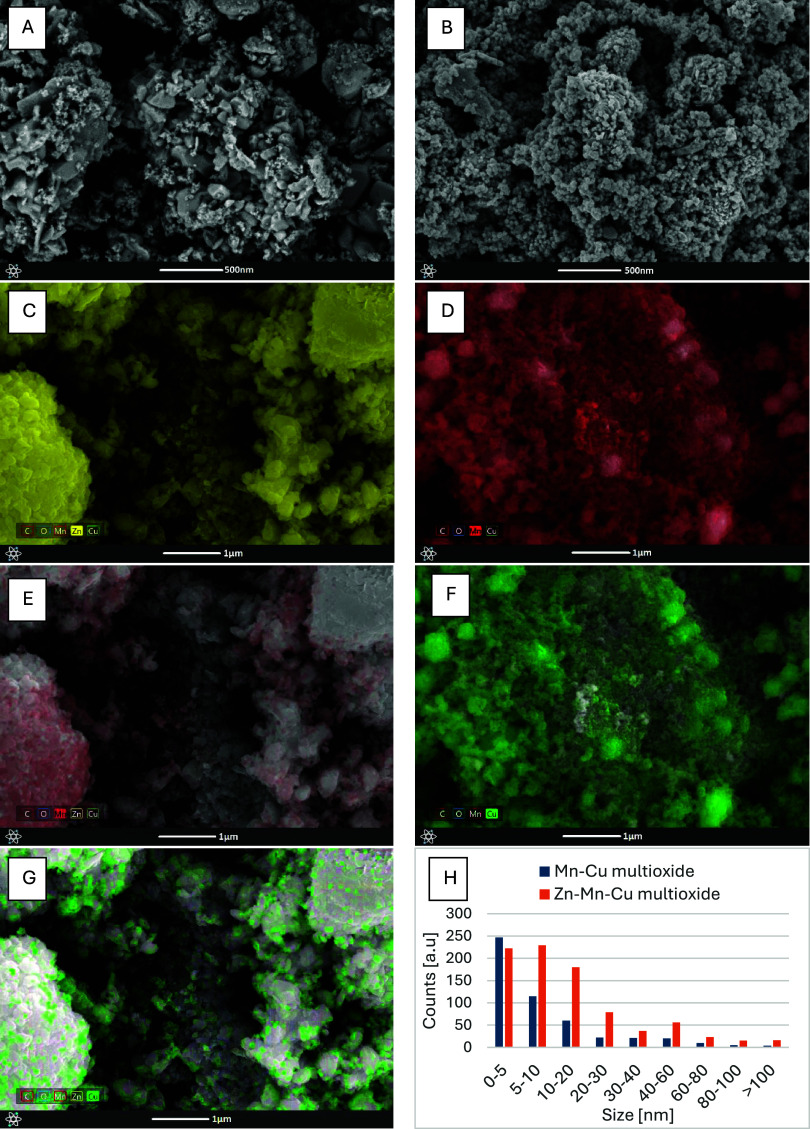
SEM photographs of materials Zn–Mn–Cu multioxide (A) and material Mn–Cu multioxide (B). EDS analysis of material Zn–Mn–Cu multioxide (C–for Zn, E–for Mn, G–for Cu) and material Mn–Cu multioxide (D–for Mn, F for Cu). Histogram (H) represents size distribution based on SEM images.

Similar characteristics can be seen for the materials obtained by the team of Mohamed et al.,^30^ who prepared Mn_3_O_4_/ZnO and CuO/ZnO-containing materials from chloride precursors by coprecipitation using a hydrothermal process.

### 2.2 Determination of Enzymatic Activity of Nanomaterials

Biocatalytic assays were performed to investigate the enzymatic activity of the tested nanomaterials as potential nanozymes. The nanomaterials were compared with two groups of enzymes: hydrolases (bromelain) and oxidoreductases (peroxidase). The mechanism of action of these biocatalysts is different, so the choice of bioactivity assays allowed conclusions to be drawn about the potential mechanism of action of the nanozymes. The results helped to assign them a potential enzymatic reaction mechanism and identify different biocatalytic behaviors.

#### 2.2.1 ABTS Method: Antiradical Activity

Each of the synthesized materials showed antiradical activity against the ABTS radical, which could be compared with the results for the pure enzymes peroxidase and bromelain (Figure 3). Materials showed activity expressed as Trolox concentration in μmol/mL: 377.56 μmol/mL Mn–Cu multioxide, 218.56 μmol/mL Zn–Mn–Cu multioxide, 388.8 μmol/mL bromelain, 259.8 μmol/mL peroxidase. Statistical analysis confirmed the statistical importance of the results with *p* = 0.003 < α = 0.05. Zn–Mn–Cu multioxide is grouped with peroxidase with importance *p* = 0.54, and Mn–Cu multioxide is grouped with bromelain with importance of *p* = 0.188, calculated for Fisher posthoc test within ANOVA. Groups are denoted with letters *a* and *b*.

**Figure 3:**
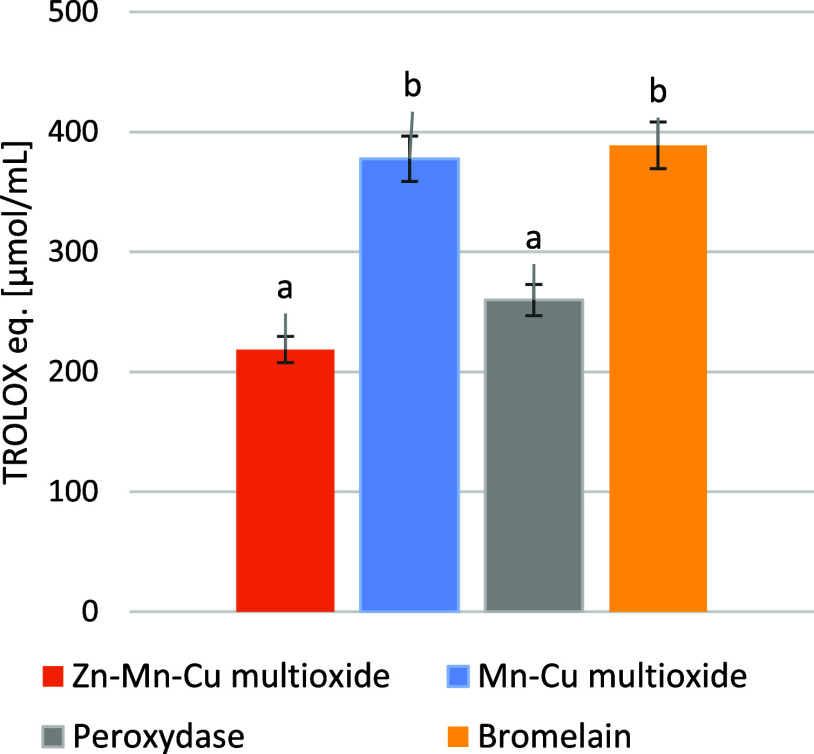
ABTS antiscavening effect of materials Zn–Mn–Cu multioxide and Mn–Cu multioxide, in comparison to two enzymes: bromelain and peroxidase, measured as Trolox equivalents in μmol/mL.

The antiradical activity of the Zn–Mn–Cu multioxide material could be compared with that of peroxidase, and that of the Mn–Cu multioxide material with that of bromelain. Compared to the homogeneous CuO and MnO oxides synthesized by Neethidevan et al.,^31^ Zn–Mn–Cu multioxide and Mn–Cu multioxide show twice the activity against the radical. In addition, Zn–Mn–Cu multioxide shows similar activity to CuO in the study of Rehana et al.,^32^ and Mn–Cu multioxide is twice this value.

#### 2.2.2 Proteolytic Assay–Hydrolase-like Activity

The proteolytic activity assay (Figure 4) was used to determine the direction of contaminant degradation by nanomaterials according to the mechanism of enzymatic hydrolysis. Bromelain and the extract containing this enzyme belong to the group of hydrolases - enzymes that degrade the substrate by hydrolysis. These substances were used as a reference to measure the activity of the materials. Zn–Mn–Cu multioxide did not show proteolytic activity, due to the presence of zinc. Mn–Cu multioxide, probably due to its higher manganese oxide content, showed activity at 0.2 U/mL. The pure bromelain enzyme showed an activity of 0.45 U/mL. The commercial extract showed an activity of 0.25 U/mL, approximately 48% compared to pure bromelain. The Mn–Cu multioxide material was capable of degrading the protein in a hydrolysis reaction. Statistical analysis confirmed the statistical importance of the results with *p* = 0.000 < α = 0.05. Zn–Mn–Cu multioxide is not grouped with any reference material. Pure bromelain is forming a group of results not related to others, and Mn–Cu multioxide is grouped with bromelain extract ZSK with importance of *p* = 0.348, calculated for Fisher posthoc test within ANOVA. Groups are denoted with letters *a*, *b*, and *c*.

**Figure 4:**
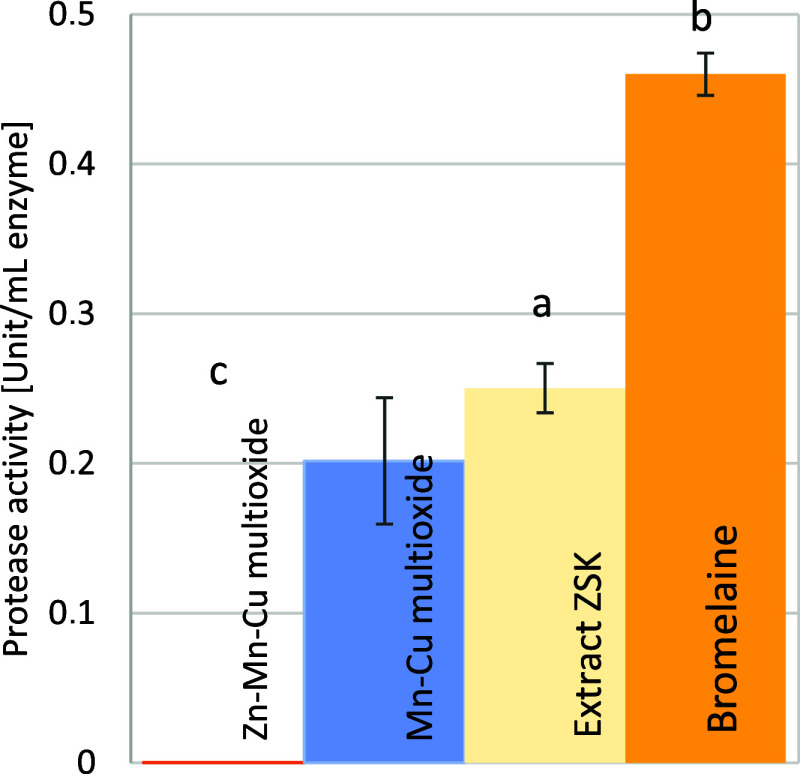
Protease activity of materials Zn–Mn–Cu multioxide and Mn–Cu multioxide in comparison with the proteolytic bromelain enzyme and commercial extract “ZSK”. Values are given in units per milliliter of enzyme. Unit is μmol of freed tyrosine per minute of reaction.

According to Kiewiet et al.,^33^ the reaction catalyzed by enzymes from the hydrolase group can be represented as given in the following equation:
1

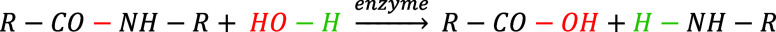




#### 2.2.3 Peroxidase Assay–Oxidoreductase-like Activity

Further analysis was followed by a peroxidase activity assay (Figure 5) involving the oxidation of guaiacol in the presence of hydrogen peroxide. The activity of Zn–Mn–Cu multioxide was twice as high as that of the Mn–Cu multioxide material: it was 0.1 U/mL versus 0.04 U/mL. Statistical analysis did not confirm the statistical importance of the results with *p* = 0.063 > α = 0.05. Zn–Mn–Cu multioxide and Mn–Cu multioxide results are not related to each other.

**Figure 5:**
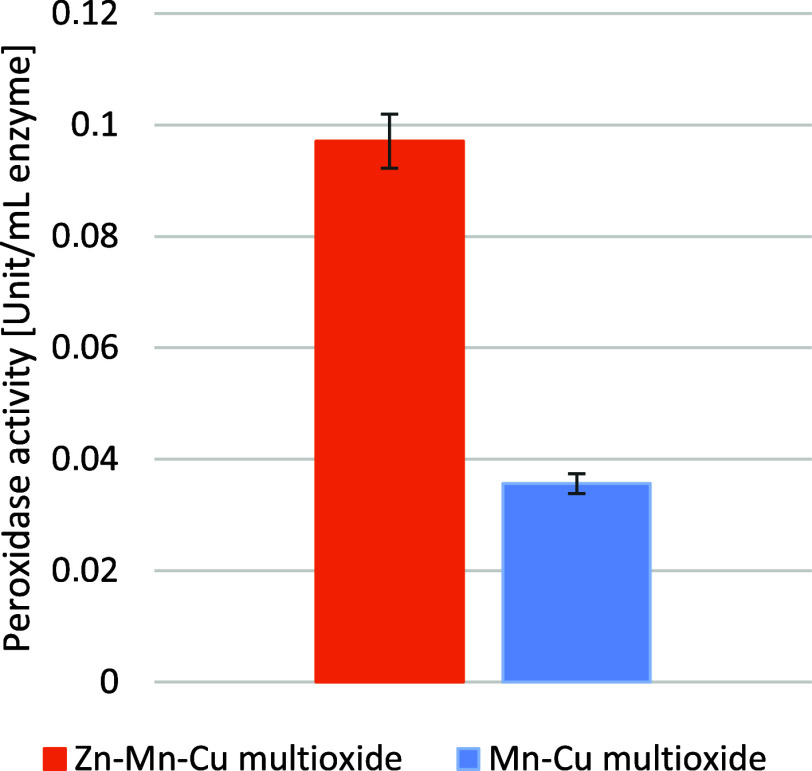
Peroxidase activity of Zn–Mn–Cu multioxide and Mn–Cu multioxide measured as unit/mL of the peroxidase enzyme.

In a study by Alzahrani and co-workers,^34^ the activity of immobilized horseradish peroxidase on CuO on the SDS polymer matrix was 0.2 U/mg, with 2% Cu addition.

Enzymes of the peroxidase group help electron transfer from the donor molecule to hydrogen peroxide, leading to the oxidation of the donor molecule and the reduction of hydrogen peroxide to water. According to Oliveira and co-workers,^35^ the electron transfer occurs at the active center of the enzyme and, importantly, two electrons are generated during the reaction, unlike the Fenton reaction, after which the enzyme returns to the ground state. The reaction can be schematically represented as
2

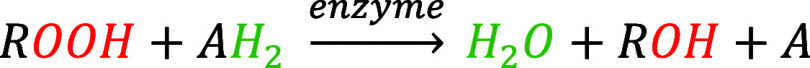

where A is the electron donor in the oxidized form, or as decomposition of H_2_O_2_ if no other substrate is present:

$$2H_{2}O_{2}\overset{\mathrm{enzyme}}{\to }2H_{2}O+O_{2}$$

### 2.3 Studies on the Photocatalytic and Biocatalytic Activity of Nanomaterials

After the investigation of possible reaction mechanisms, the photocatalytic properties of the nanomaterials were examined. The decomposition efficiency of the trypan blue dye in the presence of UV-light materials and under vis-LED light was compared. Then, combining enzymatic activities in the catalysis, the decomposition of the dye in the presence of H_2_O_2_ was carried out, verifying the applicability of nanomaterials as nanozymes for the decomposition of environmental pollutants.

#### 2.3.1 Photocatalytic Decomposition

Photocatalytic activity was tested after 30 min of dye sorption in the dark conditions, and then the TB decomposition for each material was measured at 15 min intervals (Figure 6). The decomposition by material Zn–Mn–Cu multioxide exceeded 70% and by material Mn–Cu multioxide 50%, after 1 h of light irradiation. For the reference material ZnO, the decomposition was over 90%. The results for these materials are higher than for the ZnO-Tb material used by Ghrib et al. (48%).^36^ However, according to the study by Migdadi et al.^37^ for the ZnO/CuO material, containing 10% mass copper addition, the photocatalysis of the dye reached 97% after 90 min of irradiation, which could suggest an extended irradiation process.

**Figure 6:**
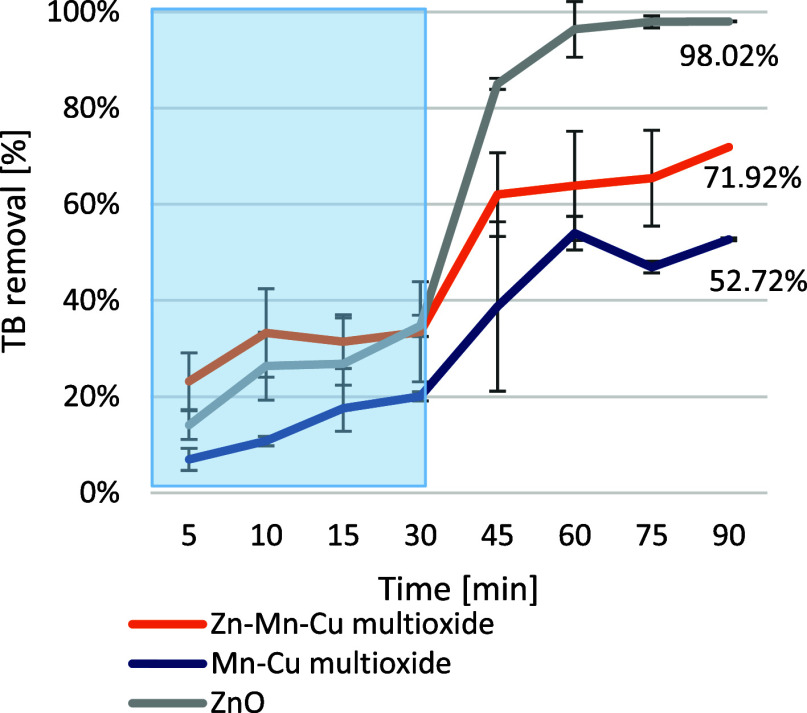
Photocatalysis activity of ZnO, Zn–Mn–Cu multioxide, and Mn–Cu multioxide, after 30 min of the dark process followed by 60 min irradiation by UV light.

The materials were also examined under vis-LED light imitating sunlight (Figure 7) to test the feasibility of the nanomaterials in decomposing the dye in an aqueous environment without the use of an artificial light source. The Mn–Cu multioxide material showed the best activity under vis-LED light, reducing trypan blue by more than 45%. The material Zn–Mn–Cu multioxide and pure ZnO oxide achieved dye reductions of 38 and 32%, respectively. As reported by Cabelo-Guzmán et al.,^38^ modification of ZnO with copper ions can change the energy levels between the valence band and the conduction band, which narrows the value of the ZnO energy gap. In the present study, this is evident because Zn–Mn–Cu multioxide was more active in the vis-LED light than pure ZnO material. In the study,^27^ it was proven that the ternary catalyst (CuO/Mn3O4/ZnO) achieved the highest degradation efficiency of Rebeprazole (97%) compared to the dual and single systems. The complementary energy gaps between phases allowed it to be used efficiently in visible light. Similarly, in both nanozymes, the use of Cu and Mn metals improved the materials’ activity in visible light. Alzahrani’s study^39^ used a cerium catalyst with a CuMn_2_O_3_ phase, where it was proven that the energy gap is comparable to that of CuO. In addition, such a gap will interact with that from ZnO, improving the efficiency of the Zn–Mn–Cu multioxide catalyst and enabling its operation under vis-LED light. Additionally, based on studies of Zhao et al.^40^ and Bayat and Sheibani,^41^ it can be assumed that the conjunction of manganese and copper oxides results in material active in vis-LED light, as these studies show, Mn–Cu multioxide. In the literature, materials of CuO doped with zinc or iron can be found, resulting in approximately 40% decomposition of the MB dye under visible light mimicked by a xenon light source, developed by George et al.^42^

**Figure 7:**
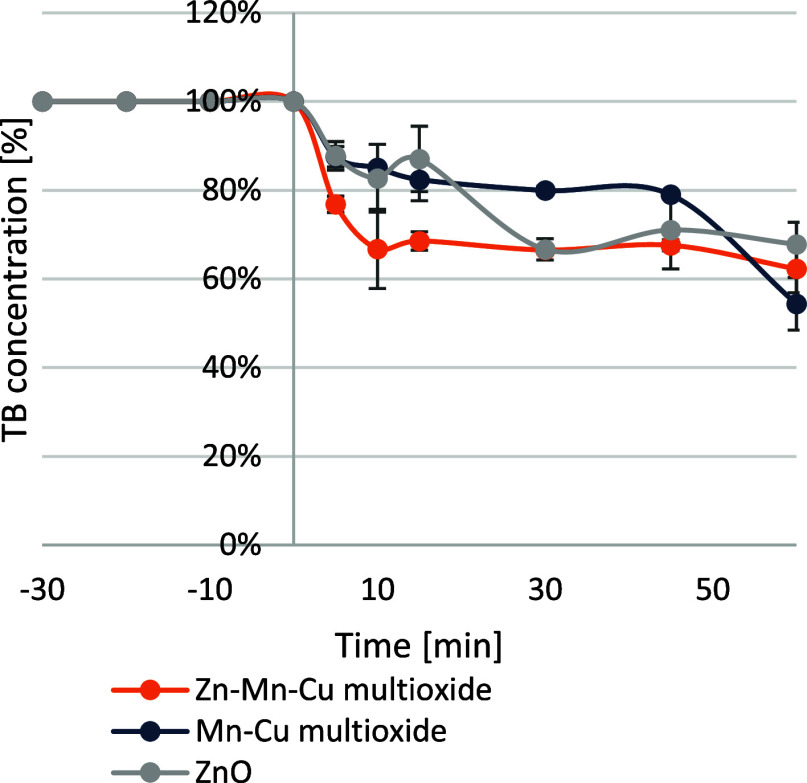
Photocatalysis in LED light (visible light imitation) for nanomaterials Zn–Mn–Cu multioxide and Mn–Cu multioxide and ZnO as reference, presented as a decrease in trypan blue concentration [%].

In addition, the materials were assessed in repeated 15 min photocatalytic cycles under UV light (Table 2). In the first cycle, Zn–Mn–Cu multioxide could be compared with ZnO, reducing the amount of dye by 60% and Mn–Cu multioxide by 47%. In the next two cycles, the activity of materials Zn–Mn–Cu multioxide and Mn–Cu multioxide remained at a similar level, degrading the dye by about 20% of the initial value. The activity of ZnO also decreased, however, it retained more activity than the nanozymes. Compared to the material consisting of Fe_2_O_3_ obtained by the team of Fattahi et al.,^43^ the cyclic use of nanomaterials also showed a decrease in catalytic activity by nearly half (74 to 47%, over two cycles). According to the authors of the study, this is a negative feature that should be moderated in future studies.

**Table 2:** Trypan Blue Decomposition after Each Photocatalytic Cycle for Materials in Comparison to ZnO^a^

material	Zn-nanoxide	Mn-nanoxide	ZnO
Cycle 1	64%	47%	62%
Cycle 2	22%	21%	48%
Cycle 3	20%	18%	40%

a Cycles were conducted after a 30 min period of dark process and 15 min UV irradiation.

#### 2.3.2 Biocatalytic-like Decomposition

The next stage was to use enzymatic activity to decompose the TB dye in the presence of hydrogen peroxide, as shown in Figure 8. For the materials tested, the addition of peroxide resulted in decomposition of the dye by 24% for the Zn–Mn–Cu multioxide material and 27% for the Mn–Cu multioxide. The control sample with only H_2_O_2_ added to the reaction showed only a 5% reduction in TB dye throughout the whole experiment time and was not included in the figure. A crucial element in the study was the absence of an additional light source to affect activity. The biocatalytic activity achieved is only due to the enzyme-like behavior of the nanomaterials. In the study by Kalsoom et al.,^44^ the degradation of trypan blue by soybean peroxidase entrapped in polyacrylamide was carried out. The activity of the system was verified for an enzyme of 40 U/mL activity and a dye concentration of 20 ppm. The remaining concentration of the dye after the first cycle of the experiment was close to 30%. However, the concentration of the used enzyme (soy peroxidase) was 40 times higher than the calculated activity of the nanozymes in this research. Compared to the nanozymes assessed in the current research, the enzyme-mediated decomposition took a shorter time, yet the amount of used enzyme was much higher and would be more expensive than the use of the study's enzymes.

**Figure 8:**
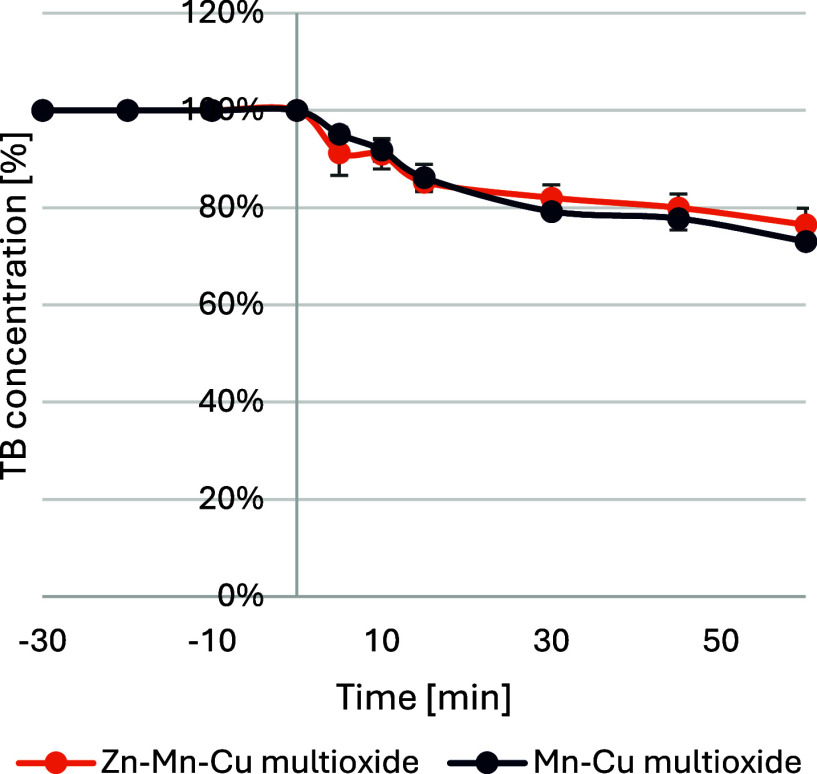
Biocatalytic-like decomposition for nanomaterials Zn–Mn–Cu multioxide and Mn–Cu multioxide, as a decrease in concentration of TB [%] with the addition of H_2_O_2_.

The addition of H_2_O_2_ enabled enzymatic decomposition of the dye according to the reaction:
3

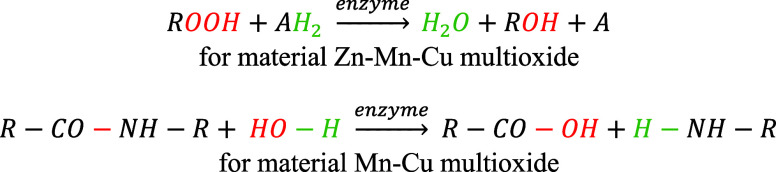




Similar mechanisms could be observed in natural enzymes, as shown in the article by Kalsoom et al.,^44^ where MS/LS studies confirmed cleavage of the dye at the −NH–C– bond and further decomposition with aromatic ring opening and −OOH group break. This mechanism occurs in peroxidases and would be more probable for the Zn–Mn–Cu multioxide. However, the mechanism used by Mn–Cu multioxide is naturally occurring for hydrolase-imitated action where the −NH–C– bond is “attacked” as a first one. Additionally, for both of the nanomaterials, a Fenton-like decomposition approach could be used. This mechanism was presented in the research of Kar et al.,^45^ where trivalent ion is reduced into bivalent ions, with simultaneous formation of hydroperoxyl radicals (HO_2_^·^), created by decomposition of H_2_O_2_. Additionally, bivalent ferrous ions are oxidized at the same time, forming trivalent ions and hydroxyl radicals (OH^·^). In the end, dye is decomposed, and radical addition takes place, and other radicals are decomposed into O_2_ and H^+^. The nanozymes in this study contain both trivalent and bivalent ions within their structures, which can promote reactive oxygen species formation. In the presence of H_2_O_2_, Mn and Cu ions in the nanozymes facilitate the conversion of H_2_O_2_ into these radicals, which then react with organic substrates, leading to the formation of hydroxylated products (ROH) or degraded products (R^·^). These mechanisms mimic natural enzyme activity, enabling nanozymes to catalyze the degradation of organic compounds in environmental applications.

## 3 Conclusions

In the present study, Zn–Mn–Cu multioxide and Mn–Cu multioxide nanomaterials were synthesized and characterized as materials combining the properties of nanozymes and photocatalysts, and mechanisms for the degradation of trypan blue dye were proposed. Using the ABTS radical reduction reaction and comparing the activity of the materials to pure enzymes, it was possible to determine the initial similarity of the nanomaterials to selected biocatalysts with antiradical activity. Determination of proteolytic activity levels allowed the determination of mechanisms for the degradation of aqueous contaminants by hydrolysis. The peroxidase activity allowed further characterization of the nanomaterials, bringing conclusions about the bioactivity of the nanozymes closer. The selection of biocatalytic methods allowed the determination of the similarity and association of the nanomaterial activity with the corresponding enzyme group of hydrolases (Mn–Cu multioxide) or oxidoreductases (Zn–Mn–Cu multioxide), as well as an approximation of the reaction mechanism of the biocatalytic degradation of trypan blue dye.

Zn–Mn–Cu multioxide appears to be a versatile nanomaterial with nanozyme and photocatalytic activity, due to the simultaneous presence of a zinc oxide phase used for photocatalysis and a CuMn_2_O_4_ spinel phase, two metals found as active centers for enzymes.^46,47^ The activity of the Zn–Mn–Cu multioxide material was similar to that of the peroxidase enzyme, as confirmed by the activity toward the ABTS radical (218.6 μmol/mL for the nanozyme and 259.8 μmol/mL for the pure enzyme), which was also confirmed in statistical analysis. The peroxidase activity for Zn–Mn–Cu multioxide was twice that of Mn–Cu multioxide at 100 mUnit/mL. The materials resembled enzymes from the oxidoreductase (Zn–Mn–Cu multioxide) or hydrolase (Mn–Cu multioxide) groups, respectively, to propose a biocatalytic mechanism for the decomposition of the dye.^35^ The photocatalytic activity under ultraviolet light for Zn–Mn–Cu multioxide was 20% higher than that of Mn–Cu multioxide due to the presence of zinc. Under mimicked visible light, the zinc-containing material was active due to the presence of copper and manganese. As reported by Ebrahimi et al.,^48^ doping zinc oxide with other metals allows for the reduction of the energy gap in the oxide structure, thus broadening the absorption spectrum from ultraviolet to visible light. Nevertheless, Mn–Cu multioxide exhibited a higher photocatalytic activity under vis-LED light, achieving 46% degradation of trypan blue. Based on the research of Zhao et al.^40^ and Bayat and Sheibani,^41^ it can be concluded that the combination of copper and manganese oxides gives a good catalyst active in LED light. Biocatalysis-like studies using H_2_O_2_ as a substrate did not show significant differences between the materials, achieving approximately 25% degradation of the initial dye concentration. In the biocatalysis-like reaction, Mn–Cu multioxide may first reduce H_2_O_2_ and then use the resulting water to further hydrolyze the dye, due to the higher antiradical activity and different mechanisms of action.^33^ Additionally, the Mn–Cu multioxide nanomaterial also showed good catalytic activity under UV light and activity as a nanozyme, which can be useful in catalysis.

Nanozymes offer unique advantages due to their combined biocatalytic and photocatalytic properties, making them versatile tools for various applications. The Zn–Mn–Cu and Mn–Cu multioxide nanozymes exemplify this dual functionality, demonstrating enzyme-mimicking activity through mechanisms resembling hydrolases or oxidoreductases. Nanozymes’ ability to catalyze reactions across a range of conditions is a good direction for further industrial-oriented research. Additionally, these nanozymes exhibit high photocatalytic efficiency under ultraviolet and visible light, as the incorporation of biologically active metal ions such as manganese and copper enhances catalytic performance. This synergy of properties highlights their potential in environmental remediation, particularly in degrading pollutants such as dyes, through both biocatalysis and light-driven processes.

While these materials show great promise, optimizing their activity under visible light and ensuring stability in complex environmental conditions could further enhance their practicality. By focusing on advanced surface modifications or doping strategies, we can refine their selectivity and long-term reusability. Further studies using the dual activities of nanozymes may bring more solutions in photobiocatalysis of environmental pollutants, paving the way for even more effective, durable, and sustainable applications.

## 4 Methods

### 4.1 Materials

Copper­(II) sulfate pentahydrate CuSO_4_·5H_2_O (Sigma-Aldrich), zinc­(II) chloride ZnCl_2_ (Sigma-Aldrich), and manganese­(II) sulfate monohydrate MnSO_4_·H_2_O (Sigma-Aldrich) were used as oxide nanoparticle precursors. Sodium hydroxide NaOH (Sigma-Aldrich) was used as a precipitating agent. The enzymes used as reference material were peroxidase from horseradish (Sigma-Aldrich) and bromelain from the stem (ThermoScientific); additionally, pineapple extract from a cosmetic company was used as an enzymatic reference (ZróbSobieKrem–“ZSK”). Materials used in activity tests and sorption were: trichloroacetic acid (TCA) (Sigma-Aldrich), 6-hydroxy-2,5,7,8-tetramethyl-chromane-2-carboxylic acid (TROLOX) (Sigma-Aldrich), Folin–Ciocâlteu phenolic reagent (Sigma-Aldrich), casein (Sigma-Aldrich), 2,2-diphenyl-1-picrylhydrazyl (DPPH) (Sigma-Aldrich), 2,2’-azino-bis­(3-ethylbenzothiazoline-6-sulfonic acid (ABTS) (Sigma-Aldrich), albumin from bovine (Sigma-Aldrich), hydrogen peroxide (POCH), guaiacol (Sigma-Aldrich), and trypan blue (TB) (Sigma-Aldrich).

### 4.2 Synthesis of Nanomaterials

A coprecipitation method was used to obtain the nano-oxides. The multimetallic nanooxides were labeled as ‘Zn–Mn–Cu multioxide’ and ‘Mn–Cu multioxide’. The synthesis was carried out in a MAGNUM II microwave reactor in a Teflon vessel.

Aqueous solutions of copper­(II) sulfate, manganese­(II) sulfate, and zinc­(II) chloride, and a solution of NaOH as a precipitating agent were prepared. Zinc­(II) chloride solution (*C* = 3.5 mol/L, *V* = 4.82 mL) was added directly to the Teflon vessel and precipitated with NaOH solution (*C* = 3 mol/L, *V* = 14.52 mL). Then, copper sulfate (*C* = 0.62 mol/L, *V* = 5.69 mL) and manganese sulfate (*C* = 0.96 mol/L, *V* = 6.00 mL) solutions were dropped simultaneously while stirring in a Hielscher UP400 St sonicator. The systems were homogenized for 5 min.

Similarly, bioxide material was prepared, where solutions of copper sulfate (*C* = 1 mol/L, *V* = 6.00 mL) and manganese sulfate (*C* = 3.5 mol/L, *V* = 4.82 mL) were added directly to the Teflon vessel and precipitated with NaOH solution (*C* = 3 mol/L, *V* = 14.52 mL) while simultaneously stirring in a Hielscher UP400 St sonicator. The systems were homogenized for 5 min.

The last step was identical for both materials. The vessel with a total volume of 30 mL was then placed in a MAGNUM II microwave reactor, and the process was carried out at *t* = 10 min, *T* = 180 °C and *p* = 20 bar. After the process, the material was filtered on 0.2 μm nitrocellulose strainers on a vacuum set and then dried for 24 h at 70 °C. After drying, the material was crushed in an agate mortar.

### 4.3 Characterization of Nanomaterials

The morphology of the resulting metal oxide compounds was investigated by scanning electron microscopy with energy-dispersive spectroscopy (Hitachi TM 3000). Based on the obtained microphotographs, particle size distribution was analyzed with the use of the ImageJ application. EDS mapping was performed for the visual and qualitative approach.

The crystallites of the nanoparticles were analyzed by structural X-ray diffraction (XRD) using a Philips X’Pert Camera diffractometer with a PW 1752/00 CuKa monochromator in the 2θ angle range, from 10 to 80°. In addition, the size of the crystallites was calculated from the Scherrer equation:

$$d=\frac{K\cdot \lambda }{\mathrm{FWHM}\cdot \cos\;\theta }$$

where *d* is the average size of crystallites, fwhm–peak width at half of its height, proportional to the size of the crystallite; *K* is Scherrer’s constant; λ is the X-ray wavelength; and θ is the angle formed by radiation with the atomic plane. The constant *K* was selected based on the shape of the particle as 0.89. Compositions of nanomaterials were calculated based on XRD results.

### 4.4 Determination of Enzymatic Activity of Nanomaterials

Following the physicochemical characterization of the materials, enzymatic activity determination methods were used to confirm the nanozymatic activity of the materials tested. Antiradical activity with the ABTS radical determined the ability to reduce the radicals formed during decomposition. The nanomaterials were tested for mimicking proteolytic hydrolase enzyme activity by decomposition of protein chains in an aqueous environment, which provided insight and the ability of nanozymes to enzymatically hydrolyze environmental contaminants. The enzymatic activity of nanomaterials mimicking the activity of peroxidases used the reduction reaction of H_2_O_2_ with the simultaneous oxidation of guaiacol - verifying this activity provided an insight into the possible enzymatic reaction mechanism of nanozymes in the oxidoreduction of pollutants. Statistical importance of data was verified by ANOVA and posthoc Fisher and Tukey tests with *p* values < α = 0.05. The importance of data and formed groups is denoted with the corresponding letters *a* and *b*.

#### 4.4.1 ABTS Method: Antiradical Activity

The ABTS method measures spectrophotometrically the reduction of the ABTS+ radical using TROLOX as a reference substance. The ABTS+ radical is generated by 2,2-azino-bis­(3-ethylbenzothiazoline)-6-sulfonic acid. This radical is a chemically stable chromophore compound with a wide pH range, which is soluble in water, and shows strong absorption in the 600–750 nm range. The antioxidants then retain the ABTS+ radical [2,2-azino-bis­(ethylbenzenothiazoline-6-sulfonic acid)], leading to a decrease in absorbance, which is detected by the antioxidant-radical combination at various times.^49^

A solution of ABTS (6 mM) was prepared with potassium persulfate (2.45 mM) in water; the solution was left in the darkroom with an aging time of 16 h. A sample (0.1 mL of enzyme or 10 mg of nanomaterial) was added to 4 mL of diluted ABTS reagent, incubated for 10 min, and then measured at 734 nm. In the assay, the nanomaterials were compared to the enzymes peroxidase and bromelain, which were diluted to similar activity. The calibration curve was based on the reaction of Trolox with ABTS reagent; the measured absorbance after the reaction corresponds to the amount of Trolox needed for the reaction expressed in μmol/mg. The curve was prepared in the range 0–40–80–120–160–200 μmol of TROLOX, and the concentration was calculated from the formula:

$$\begin{array}{l} y=-956.05x+378.95\\ R^{2}=0.9919 \end{array}$$

where *y* is the absorbance and *x* is the TROLOX concentration (μmol).

#### 4.4.2 Casein Method: Proteolytic Activity

Casein was used as a substrate to measure the proteolytic activity of the materials and the materials after immobilization. During hydrolysis of the casein protein, the reaction products are precipitated with TCA acid and then determined colorimetrically with a Folin solution, measuring the concentration of the reaction products. The calibration curve of the method is based on tyrosine, which is released during protein degradation.

A casein solution (5 mL) was added to the test sample (10 mg), and the reaction was run for 30 min at 37 °C. The reaction was then stopped with TCA acid (5 mL) and incubated for a further 30 min. The filtrate was collected and labeled with Folin solution (2 mL), incubating the mixture for 30 min. In the final step, the filtrate was collected, and its absorbance was measured spectrophotometrically at 726 nm. As reference samples, commercially available pineapple extract (labeled ZSK) and pure bromelain were used; both the extract and the pure enzyme exhibit proteolytic activity.

The calibration curve was determined in the range of 0–0.05–0.1–0.2–0.4–0.5 tyrosine concentrations. From the curve formula, the amount of released tyrosine (*x*) was calculated from the absorbance reading (*y*):

$$\begin{array}{l} y=1.1595x-0.0214\\ R^{2}=0.9977 \end{array}$$

where *y* is the read absorbance and *x* is the amount of tyrosine released (μmol).

The result was converted by the volume of the reaction mixture (*V_t_* = 11 mL) and time (*t* = 10 min), the volume of the enzyme/sample (*V*_s_ = 1 mL), and the volume after filtration (*V*_f_ = 2 mL), obtaining the final result expressed as unit/mL. The following formula was used:

$$\frac{\mathrm{unit}}{\mathrm{mL}}=\frac{x\times V_{t}}{t\times V_{s}\times V_{f}}$$

#### 4.4.3 Peroxidase Activity

Peroxidase breaks down H_2_O_2_ by converting it to water with the simultaneous oxidation of the reaction substrate. The peroxidase reaction can be monitored using guaiacol (2-methoxyphenol), which can be oxidized to produce a brown product (tetraguaiacol), which is quantified using a spectrophotometer at 420 nm. A calibration curve was prepared using peroxidase to a concentration of 0–0.1–0.5–1–1.5 U/mL, and the concentration was calculated from the formula:

$$\begin{array}{l} y=-0.0697x^{2}+0.2228x+0.0074\\ R^{2}=0.9991 \end{array}$$

Peroxidase activity was measured by the reaction of H_2_O_2_ decomposition (12 mM, 0.03 mL) by the enzyme with guaiacol (20 mM, 0.05 mL) as an indicator to change the color of the solution; 0.1 mL of enzyme or 10 mg of sample was added to the mixture, and then, after 10 min of reaction, the absorbance of the solution was measured.

### 4.5 Studies on Dye Degradation in Photocatalysis and Biocatalysis

#### 4.5.1 Photocatalytic Decomposition: UV and Vis-LED

Photocatalytic studies for nanozymes were carried out using trypan blue (TB) dye at a concentration of 50 ppm. The photocatalytic activity of the tested materials was evaluated by monitoring their ability to degrade the trypan blue dye in an aqueous environment in the presence of UV light (wavelength 364 nm, output power 12.2 W) and artificial visible LED light (intensity of light emitted 2750 lm, output power 36 W). The degradation reaction was carried out at room temperature for 60 min, sampling every 15 min. The calibration curve was based on the measurement of TB absorbance at 590 nm, in the range 0–10–20–30–40–50 ppm, and the concentration was calculated from the formula:

$$\begin{array}{l} y=0.035x+0.0481\\ R^{2}=0.9994 \end{array}$$

where *y* is the absorbance and *x* is the TB concentration (mg/L).

Based on initial tests, the dye solution (5 mL) was added to the test material samples (5 mg) and stirred for 30 min in a dark room to achieve adsorption and desorption equilibrium. The reaction mixture was then exposed to ultraviolet (UV) or visible light (LED-vis) to study the photocatalytic degradation of the dye in the presence of Zn–Mn–Cu multioxide and Mn–Cu multioxide materials. After a set time (5–10–15–30–45–60 min), the material was filtered on a syringe filter (0.22 μm) and the dye concentration was monitored spectrophotometrically. The tests were carried out in duplicate. The solution of the dye itself (blank) was also photodegraded in parallel with the test samples to exclude the effect of decomposition of the dye itself. The calculated result for the nanomaterial in a given time unit was related to that of the parallel blank.

In addition, tests for the repeated use of nanomaterials in the decomposition of the dye were carried out by repeating the photocatalysis reaction three times. For the reaction cycles, samples were prepared as for the UV-induced photocatalytic activity test: 5 mL of dye and 5 mg of material were used. Each catalytic cycle lasted 15 min; after a given cycle, the nanomaterial and dye were separated by centrifugation. After 10 min of centrifugation (10,000 rpm), the dye was collected for absorbance testing, and the material used was purified with distilled water (2 mL) and separated by centrifugation before the next cycle. A new batch of dye was added to the material and thus purified.

To evaluate the performance of the cycles, the following equation was used:

$$\mathrm{Degradation}\;\mathrm{efficiency}=\left(\frac{C_{t}}{C_{0}}\right)$$

where *C*_0_ and *C_t_* are the respective dye concentrations at *t* = 0 (initial time) and 15 min, respectively.

#### 4.5.2 Biocatalytic-like Decomposition

Biocatalytic-like tests were carried out using trypan blue (TB) dye at an initial concentration of 50 ppm and the addition of H_2_O_2_ for a 60 min reaction time. The study was designed to investigate the effect of the enzymatic degradation of the dye by the materials and to check the enzymatic mechanism of the reaction. The degradation reaction was carried out at room temperature and without an additional light source. The calibration curve was based on the measurement of TB absorbance at 590 nm, in the range 0–10–20–30–40–50 ppm, and the concentration was calculated from the formula:

$$\begin{array}{l} y=0.035x+0.0481\\ R^{2}=0.9994 \end{array}$$

where y is the absorbance and x is the TB concentration (mg/L)

The dye solution (5 mL) was added to a sample of material (5 mg), H_2_O_2_ (0.042%, 0.25 mL) was added, and the reaction was run for a set time (5–10–15–30–45–60 min); then the material was filtered on a syringe filter (0.22 μm), and the dye concentration was monitored spectrophotometrically. The control sample with only H_2_O_2_ was prepared in the same manner. The study was carried out in duplicate. To evaluate the degradation efficiency, the equation used was

$$\mathrm{Degradation}\;\mathrm{efficiency}(\%)=\frac{\left(C_{0}-C_{t}\right)}{C_{0}}\times 100$$

where *C*_0_ and *C_t_* are the respective dye concentrations at *t* = 0 (initial time) and *t* = set time [min].

## Data Availability

Not applicable or available upon request.
